# Comparative genomics of *Pseudomonas paraeruginosa*

**DOI:** 10.1128/jb.00149-25

**Published:** 2025-07-25

**Authors:** Maxime Déraspe, Lori L. Burrows, Romé Voulhoux, Daniela Centrón, Jacques Corbeil, Paul H. Roy

**Affiliations:** 1 Infectious and Immune Diseases Research Program, CHU de Québec-Université Laval Research Center, Québec City, Québec, Canada; 2 Department of Molecular Medicine, Faculty of Medicine, Université Laval 12369 , Québec City, Québec, Canada; 3 Department of Biochemistry and Biomedical Sciences, and the Michael G. DeGroote Institute for Infectious Disease Research, McMaster University 536887, Hamilton, Ontario, Canada; 4 LCB-UMR7283, CNRS, Aix Marseille Université, IMM 128791, Marseille, France; 5 Universidad de Buenos Aires, Consejo Nacional de Investigaciones Científicas y Tecnológicas, Instituto de Investigaciones en Microbiología y Parasitología Médica (IMPAM), Facultad de Medicina, Buenos Aires, Argentina; 6 Department of Biochemistry, Microbiology and Bio-informatics, Faculty of Sciences and Engineering, Université Laval 98637, Québec City, Québec, Canada; National Institutes of Health, Bethesda, Maryland, USA

**Keywords:** *Pseudomonas*, genomics, antibiotic resistance, virulence

## Abstract

The PA7-clade (or group 3) of *Pseudomonas aeruginosa* is now recognized as a distinct species, *Pseudomonas paraeruginosa*. We report here the genomic sequences of six new strains of *P. paraeruginosa*: Zw26 (the first complete genome of a cystic fibrosis isolate of *P. paraeruginosa*)*,* draft genomes of four burn and wound strains from Argentina very closely related to PA7, and of Pa5196, the strain in which arabinosylation of type IV pili was documented. We compared the genomes of 82 strains of *P. paraeruginosa* and confirmed that the species is divided into two sub-clades. Core genomes are very similar, while most differences are found in “regions of genomic plasticity” (RGPs). Several genomic deletions were identified, and most are common to the CR1 sub-clade that includes Zw26 and Pa5196. All strains lack the type 3 secretion system (T3SS) and instead use an alternative virulence strategy involving an exolysin, a characteristic shared with group 5 *P*. *aeruginosa*. All strains tend to be multiresistant like PA7, with a significant proportion of carbapenem-resistant strains, either *oprD* mutants or carrying carbapenemase genes. Although *P. paraeruginosa* is still relatively rare, it has a worldwide distribution. Its multiresistance and its alternative virulence strategy need to be considered in future therapeutic development.

**IMPORTANCE:**

*Pseudomonas aeruginosa* is an important opportunistic pathogen causing respiratory infections, notably in cystic fibrosis, and burn and wound infections. Our study reports six new genomes of *Pseudomonas paraeruginosa*, a new species recently reported as distinct from *P. aeruginosa*. The number of sequenced genomes of *P. paraeruginosa* is only about 1% that of *P. aeruginosa*. We compare the genomic content of nearly all strains of *P. paraeruginosa* in GenBank, highlighting the differences in core and accessory genomes, antimicrobial resistance genes, and virulence factors. This novel species is very similar in environmental spectrum to *P. aeruginosa* but is notably resistant to last-line antibiotics and uses an alternative virulence strategy based on exolysin—this strategy being shared with some *P. aeruginosa* outliers.

## INTRODUCTION

The genome sequence of a *Pseudomonas aeruginosa* taxonomic outlier, PA7, was reported in 2010 (1). This non-respiratory clinical isolate was notable for its multiresistance, novel genomic islands, and lack of a type 3 secretion system (T3SS). Sequencing of some housekeeping genes showed that PA7 was closely related to the known taxonomic outlier DSM1128 (ATCC9027) (1), an outer ear isolate from Australia. Studies on another related strain, Pa5196, a rectal isolate from Canada, showed that its type IV pilin is glycosylated (2, 3). The glycosylation of the type IV pilins in this species with oligomers of D-arabinofuranose (D-Araf) is one of its distinctive traits and confers resistance to pilus-specific bacteriophages (4, 5). In other bacteria such as *Acinetobacter* or *Neisseria*, this form of post-translational modification masks the pili from antibody recognition and is thus involved in immune evasion (6). Type IV pilins in *P. aeruginosa* can also be glycosylated, but the enzyme responsible is different, and the glycan is the LPS O antigen (7). A novel virulence factor, exolysin, was described in strains CLJ1 and CLJ3, tracheal aspirate isolates from France (8), and mediates a high level of cytotoxicity in the former strain. This factor can compensate for the lack of the T3SS.

Most of the accessory genomes of *P. paraeruginosa*, like *P. aeruginosa*, occur as insertions into “regions of genomic plasticity” (RGPs), first described by Mathee et al. (9). Novel RGPs were found by Roy et al. in PA7 (1), and the lists were expanded by Klockgether et al. (10) and by Sood et al. (11). RGPs typically contain prophages, restriction–modification systems, antibiotic and heavy metal resistance, transport and secretion systems, and supplementary metabolic pathways. RGPs were defined as insertions of at least four genes. Parts of the accessory genome, e.g., the exolysin genes, are in smaller insertions.

The PA7 clade was previously considered to be *P. aeruginosa* based on its rRNA sequence. Recently, Rudra et al. (12) proposed that the PA7 clade be considered as a new species, *Pseudomonas paraeruginosa*, with DSM1128 (ATCC9027) as the type strain, based on current criteria including average nucleotide identity, average amino acid identity, and digital DNA–DNA hybridization values.

*P. paraeruginosa* is characterized by its similarity to the first completely sequenced genome, that of PA7, isolated in Argentina in 2010. We report here the complete genome sequence of *P. paraeruginosa* Zw26, a cystic fibrosis strain isolated in Germany, and five draft genome sequences: burn strains pae802, pae815, and pae832; wound strain pae413, all from Argentina and closely related to PA7; and Pa5196, a rectal isolate from Canada. Using NCBI TBLASTN with a 200 nt segment from the *dnaA* gene as the query and the genus *Pseudomonas* as the target, we found a total of 82 strains (seven complete genomes and 75 draft genomes) identified as *P. paraeruginosa*. Table S1 lists the origins and distribution of the sequenced strains. Most are human isolates, but several are environmental. The strains have a worldwide distribution. We constructed a phylogenetic tree and confirmed that this species is divided into two sub-clades. We provide a table that compares the predicted proteins of PA7 to all the other strains. We also provide a database of all contigs from all strains that permits rapid (<1 min) searches with nucleotide or protein query sequences.

## RESULTS

### General features of *Pseudomonas paraeruginosa*

We identified the six strains submitted in the present study, as well as 75 others, as PA7-like based on similarity to the segment (nt 701–900 of CP000744) of the *dnaA* gene, which encodes the chromosomal replication initiator protein DnaA and shows the greatest difference from *P. aeruginosa*. This region is a discriminator for *P. paraeruginosa*, with a minimum of 99% identity and 90% query coverage with TBLASTN, whereas other *Pseudomonas* spp. have a maximum of 24% query coverage. Because of the relatively low average nucleotide identity (ANI) between *P. paraeruginosa* and *P. aeruginosa,* there are potentially hundreds of other possible discriminators. Rudha et al. (12) listed 34 strains based on a conserved insertion into the *dgcB* gene, and the strains from their list are included in ours. However, we found that the same insertion also occurs in *P. aeruginosa* outliers of Freschi et al.’s “group 4” (13). The PA7 clade was referred to by Rudha et al. as “Clade 2,” while Freschi et al. referred to it as “group 3”. *P. paraeruginosa* notably lacks the T3SS and its effectors and contains the *exlBA* genes (see “Exolysin” below). However, Freschi’s *P. aeruginosa* “group 5” also has these characteristics.

**Fig 1:**
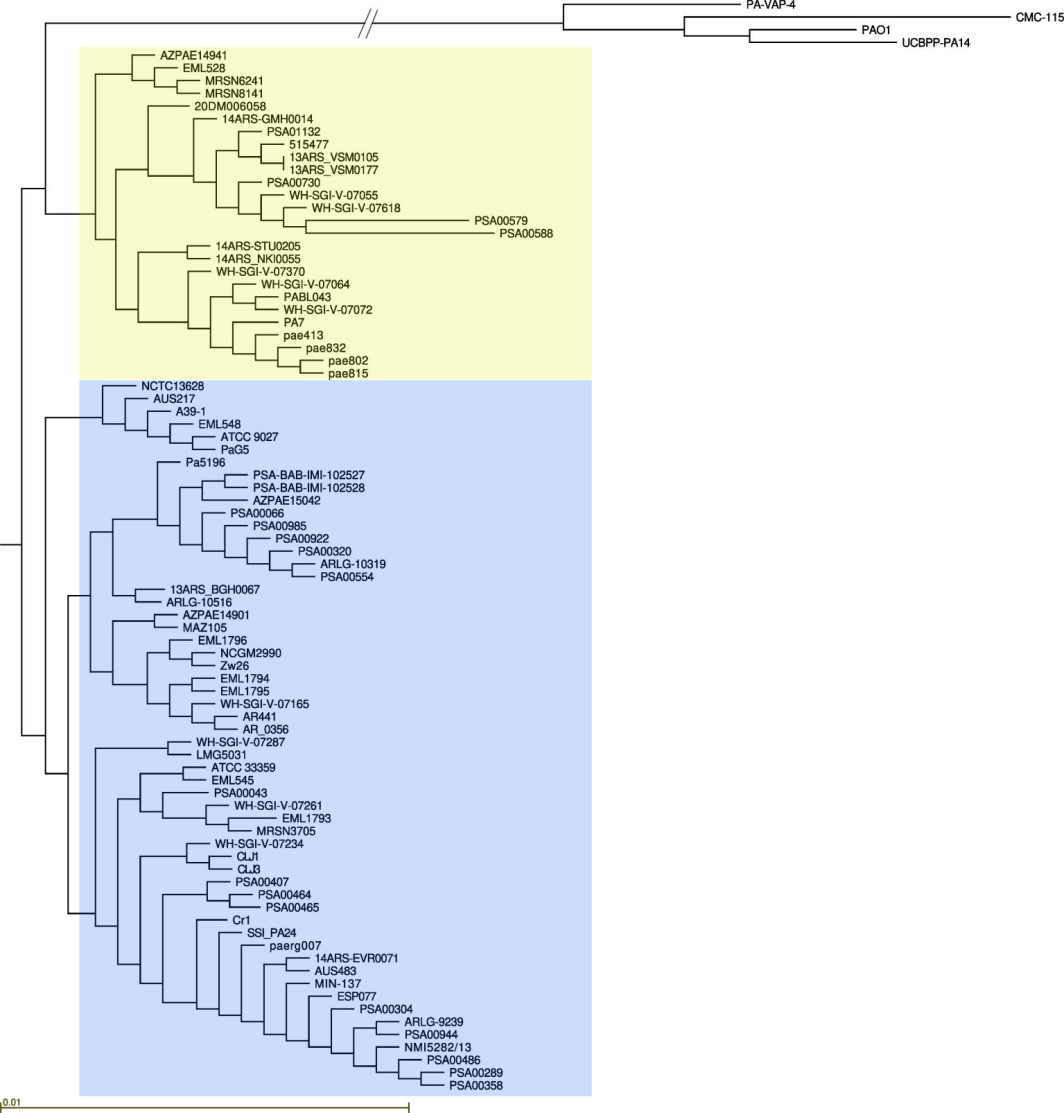
Phylogenetic tree of *P. paraeruginosa*. The tree shows that the species has two sub-clades, PA7 and CR1. The CR1 sub-clade (blue) notably has several genome reductions (Table 2).

A phylogenetic tree of *P. paraeruginosa* was constructed (Fig. 1). It shows that the species is divided into two sub-clades, one containing 26 strains, including PA7, pae802, pae815, pae832, and pae413, and the other containing 56 strains, including Zw26, Pa5196, CR1, ATCC9027, CLJ1, and CLJ3 (Fig. 1 and Table S1). This division was first found by Sood et al. (11) who sequenced the strain CR1 and analyzed 14 PA7-like strains. We analyzed the contents of 81 strains compared to PA7. The results are summarized in Table S1. A few additional strains were not included in the table due to poor sequence quality, too many contigs (>250 contigs of >500 nt), or submission to GenBank without annotation. Table S1 also includes representative strains of *P. aeruginosa* group 1 (PAO1), group 2 (UCBPP-PA14), group 4 (PA-VAP-4), and group 5 (CMC-115).

### Genome insertions

Regions of genomic plasticity (RGPs 1–62) were defined by Mathee et al. (9) as “any region of at least four contiguous ORFs that are missing in at least one of the (five) genomes analyzed” and are mostly shared between *P. aeruginosa* and *P. paraeruginosa*, although a few (e.g., RGP34) are “empty” in all *P. paraeruginosa*. RGPs 63–80, first found in PA7, are shared with *P. aeruginosa,* although the contents of eight of the 18 are exclusive to *P. paraeruginosa* (Table S1). The RGP contents of the four Argentinian draft strains are very similar to those of PA7. A few genome reductions in RGPs are noted below. In the strain Zw26, there are several differences in RGPs that are summarized in Table 1.

**TABLE 1:** RGP contents of the complete genome of Zw26 that are absent in PA7^*a*^

RGP	Zw26 locus	Number of genes	Features
66	00546–00562	17	Conjugal transfer protein TraY; insertion sequences
89	01127–01132	6	RHS family protein pseudogene; insertion sequences
58	01582–01586	5	Secreted protein Hcp
31	01787–01790	4	O antigen locus
56	02166–02177	12	DNA cytosine methylase; pirin family protein; insertion sequences
28	02246–02278	33	Phage-related
72	02369–02373	5	D-galactonate catabolism
72	02383–02449	67	Phage-related (similar phage in RGP56 in PA7)
26	02473–02480	8	Phage-related
25	02593–02599	7	ShlB family secretion protein; fragmented hemagglutinin gene
23	02792–02804	13	Phage-related
23	02816–02853	28	Pyocin, ABC and RND transporters, mercury resistance operon
Undefined	03209–03213	5	HlyD family secretion protein; ABC transporter ATP-binding protein
77	03638–03642	5	Lipoprotein
15	03653–03663	11	Threonine dehydratase; ABC transporter protein
Undefined	04177–04190	14	Phage-related
6	04278–04342	65	Phage-related; fimbrial protein
5	04436–04550	115	Integrative conjugative element
Undefined	04657–04668	12	MFS transporter
41	04877–04952	76	Integrative conjugative element
87	05572–05619	38	Phage-related
Undefined	06028–06033	6	Phage-related

^*a*^ These are part of the accessory genome; several are complete or partial prophages. Two are integrative conjugative elements.

We analyzed only the large insertions in complete genomes, and their homologs, as the contents of RGPs cannot be situated precisely in draft genomes due to contig ends. PA7 contains an integrative conjugative element (ICE) in RGP7 (PSPA7_4437-4530). ICEs with similar contents are present in the majority of strains (Table S1 and S2). The ICE in A39-1 is also in RGP7, whereas those of Zw26 and AZPAE15042 are in RGP41. A second distinct ICE appears in Zw26 in RGP5. Only two strains (EML1794 and EML1795) have similar ICEs. A third ICE mediating copper resistance, which impacts bacterial fitness, was recently described in RGP29 of AZPAE15042 (also called IHMA87) (14). This ICE is found in 11 other strains. All are human isolates. Similar ICEs without the module are found in A39-1 and seven draft genomes. A fourth ICE is found in RGP28 in AZPAE15042, with similar ICEs in 14 draft genomes (Table S2). Finally, a fifth ICE is present in RGP56 of AR441 and AR_0356.

Dit islands, containing genes that enable *Pseudomonas* strains to use abietane diterpenoids from tree resin as a sole source of carbon, are found in several *P. paraeruginosa* strains. The first Dit island described in *P. aeruginosa* 2192 (9) is composed of loci PA2G_01975–PA2G_02069 and occurs in 12 *P*. *paraeruginosa* strains with >99% similarity (Table S2, group A). Nine of these islands are in RGP29 and three in RGP27. A second group (Table S2, group B), 90% similar to the first, occurs in six strains. In strains AR441 and AR_0356, the island is in RGP6. In the other four strains, the location cannot be determined due to contig junctions. A third group (Table S2, group C), 90% similar to the other two, was described by Sentausa et al. (15) and occurred in RGP27 of CLJ1 and CLJ3. The presence of Dit islands in some human isolates supports their likely environmental origins.

Four prophages were found in the genome of PA7 (1). The PA7 RGP66 prophage is in Zw26. It is also in AR_0356 and in AR441, but located in RGP87. A 99% identical prophage (except for the integrase that is only 59% identical) occurs in PA7 in RGP78. Another prophage occurs in RGP3/RGP4 in PA7 and A39-1. Similar but defective prophages are present in the strains of *P. aeruginosa* (e.g., PAO1 and UCBPP-PA14) used to define RGPs (9). RGP3/RGP4 is in fact a single RGP. The fourth PA7 prophage, in RGP56, occurs in only four closely related draft strains. A similar prophage occurs in RGP72 of Zw26. Prophages have been associated with resistance and virulence factor genes in some pathogens (16), but this association occurs infrequently in *P. aeruginosa* (17). In *P. paraeruginosa* AR441 and AR_0356, three efflux-related transporter genes (CSC28_6032-4), possibly involved in resistance, are adjacent to the tRNA gene where a prophage is inserted in RGP87, but are not part of the insertion. Only one virulence gene, the TIR effector PSPA7_2375 (see below), was found in a prophage (RGP56) in PA7 and a few related strains.

CRISPR-Cas systems are involved in phage defense. Strain Pa5196 notably contains a type I-F CRISPR-Cas system in RGP24. This system is present in 38 *P*. *paraeruginosa* strains from four distinct branches of the phylogenetic tree (Table S2), including 10 with identical CRISPR elements (the Pa5196 branch in Fig. 1). The Cas element in all 38 is >99% identical. Two strains have type I-E systems in RGP12 that are 99.99% identical (WH-SGI-V-07287 and MG5031). Both are environmental isolates (Table S1). The genomic loci for type I-F and type I-E CRISPR-Cas systems correspond to those described for *P. aeruginosa* (18). No strains have a type I-C system.

### Genome reductions

Ten regions of the PA7 genome, not part of RGPs, are absent in all strains in the CR1 sub-clade of *P. paraeruginosa*. These include the Hcp secretion island-3 encoding the type VI secretion system (H3-T6SS), genes for several ABC transporters, and the *arnBCAD* operon (PSPA7_1593–1590) responsible for resistance to polymyxin B and cationic antimicrobial peptides. These regions are listed in Table 2. Table S4 in Sentausa et al. (15) cites them as “Specific regions of PA7 compared to CLJ,” and their list is identical to ours for all deletions not associated with RGPs.

**TABLE 2:** Genome reductions common to the CR1 sub-clade^*a*^

PSPA7 numbering	Number of genes	Features
0263–0267	4	Sulfate ester transport system
0270–0273	4	TonB-dependent receptor
0279–0281	3	*tonB2-exbB1-exbD1* region
1586–1593	8	Polymyxin and cationic antimicrobial resistance cluster
1678–1685	8	Sulfur starvation utilization operon
2884–2908	24	HCP secretion island-3 encoded type VI secretion system (H3T6SS)
2911–2916	6	Methionine ABC transporters; monooxygenases
2930–2935	6	Alkanesulfonate assimilation; nitrate and nitrite ammonification
5297–5302	6	Fimbrial chaperone/usher pathway E operon
5708–5718	11	Arginine:pyruvate transaminase; 2-ketoarginine decarboxylase

^*a*^ These blocks of non-essential genes are part of the accessory genome; they are present in strains of the PA7 sub-clade and absent in strains of the CR1 sub-clade.

Additionally, the sequences of all strains described as “CF,” “respiratory,” and “sputum” isolates were examined for strain-specific genome reductions. Zw26 had no specific genome reductions, other than those seen for the whole sub-clade, relative to PA7. While genome reductions, especially loss of virulence genes, have been described in cystic fibrosis strains where they play a role in adaptation to their environment (19), their role in other strains is less clear.

Two genomes have large reductions. Strain PSA00486 lacks PSPA7_2852–3005, including the pyoverdine locus and an adjacent region of RGP23. Strain EML528 lacks PSPA7_3074–3138, including transporters and HCN synthase. Strain MRSN8141 (20) has two shorter genome reductions, lacking PSPA7_0611–0637, which includes some heme d1 biosynthesis genes and PSPA7_3003–3019. Strain WH-SGI-V-07165 lacks PSPA7_2987–3005, while strain PSA00043 lacks PSPA7_2997–3006 (Table S1). None of these regions is considered essential for survival (21).

Our four draft burn and wound genomes belonging to strains closest to PA7 (pae802, 813, 832, and 413) have some strain-specific genomic reductions. All four strains lack the part of the contents of RGP25 (PSPA7_2776–2793), which contains a fragmented hemagglutinin gene (PSPA7_2777–2782–2787) in PA7. They also lack most of RGP78, which is related to a prophage. Strains pae802 and pae815 lack the defective prophage in RGP60 (PSPA7_5143–5157). Strain pae413 lacks PSPA7_2363–2435 (prophage in RGP56), PSPA7_3348–3383 (not an RGP, but containing two TonB-dependent transporters), and PSPA7_4205–4226 (not an RGP). Strain pae832 lacks PSPA7_2967–3055 (not an RGP but notably including the PvcABCD pyoverdine biosynthesis operon and the CupD fimbrial protein operon) (Table S1). Strain Pa5196, like other strains of the CR1 sub-clade, differs in several RGPs. It also has a deletion of the MexCD-OprJ efflux system.

### Virulence factors common to *P. aeruginosa* and *P. paraeruginosa*

**Fig 2:**
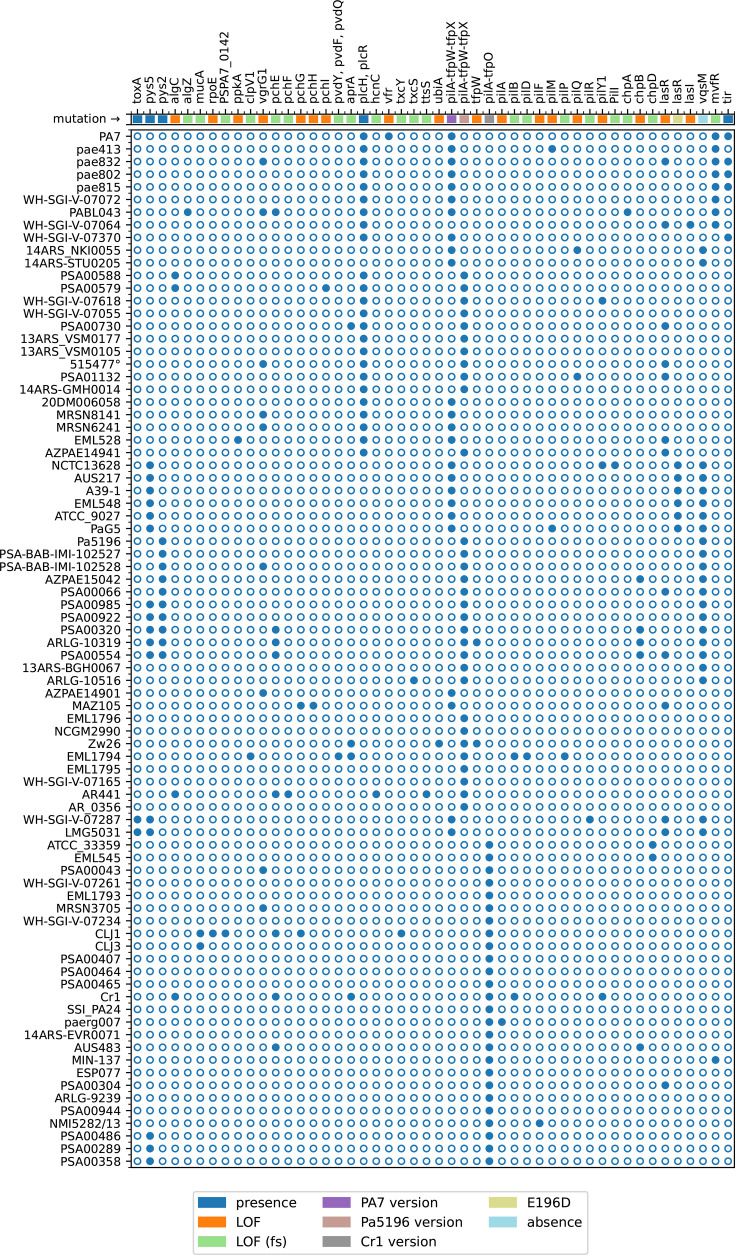
Summary of virulence genes. These are arranged in the order in which they are cited in the text. The filled circles indicate strains with the characteristics shown above.

As in PA7, all *P. paraeruginosa* lack the type 3 secretion system (T3SS) encoded by PA1690–PA1725 in PAO1. They are also missing the “T3SS translocated effectors” genes *exoS*, *exoT*, *exoU*, and *exoY*. The *toxA* gene, encoding exotoxin A (PA1148 in PAO1), is present in only two strains: WH-SGI-V-07287 and LMG5031. The *pldA* gene, which encodes the H2-T6SS-secreted effector phospholipase D, and the *rhlC* gene for rhamnolipid biosynthesis are absent in all *P. paraeruginosa* strains. However, the *rhlA* and *rhlB* genes (PSPA7_1647–1648) are present in all strains. The gene encoding pyocin S5 is present in 16 *P*. *paraeruginosa* strains (Fig. 2), but not in PA7, Zw26, or our five draft genomes. Ten strains, including Pa5196, have homologs of the *pys2* gene encoding pyocin S2.

*P. paraeruginosa* is similar to *P. aeruginosa* for most factors identified in the Virulence Factor Database (VFDB) (22). The 25 genes for biosynthesis and regulation of alginate, which is involved in biofilm formation in the CF lung, are ubiquitous, with a few exceptions: the biosynthetic genes *algC* and *algZ* and regulatory genes *mucA* and *rpoE* (*algU*) (Fig. 2). Notably, only two complete genomes (AR441 and CR1) are presumably mutated in *algC*, but in homopolymer runs (C6 for C7), so long-read sequencing errors cannot be ruled out. The 21 genes of the HCP secretion island 1 type VI secretion system (H1-T6SS) are ubiquitous and intact, except for the *vgrG1* gene, which is mutated or deleted in nine draft strains (Fig. 2).

The phenazine biosynthesis genes *phzA-phzG* occur in two copies and thus are difficult to analyze in draft genomes due to contig junctions. They are ubiquitous in complete genomes (as well as the single-copy *phzM* and *phzS* genes). Unlike *P. aeruginosa*, the *phzA1-phzB1* genes are fused (PSPA7_0888), and there is no *phzH* gene (PA0051). The PhzH protein modifies phenazine-1-carboxamide, an antifungal biopesticide. Five of the 10 pyochelin genes (*pchABCDR*), as well as the pyochelin receptor gene *fptA*, are ubiquitous in *P. paraeruginosa*. There are a few mutations, summarized in Fig. 2, in *pchEFGHI*. Among the complete genomes, the two with a supposed frameshift in *pchE* and the one in *pchF* are potentially sequencing errors (homopolymer undercounts). *P. paraeruginosa* produces type II pyoverdine (23) and has a type IIb *fpvA* gene (24). The system is intact, except for strain EML1794, which has frameshifts in *pvdY*, *pvdF*, and *pvdQ*, and strain PSA00486, which has genomic deletion mentioned above.

The alkaline protease gene *aprA* is frameshifted in four strains including Zw26 (Fig. 2). The *lasA* and *lasB* elastase genes are ubiquitous, as are the genes of the GacS/GacA two-component system. The *plcH* gene, encoding hemolytic phospholipase C, and *plcR*, encoding its accessory protein, are present in 24 of the 26 strains of the PA7 sub-clade and absent in the whole CR1 sub-clade (Fig. 1 and 2). The *plcN* and *plcB* phospholipase C genes are ubiquitous. Hydrogen cyanide production genes are ubiquitous, except for the strains EML528, where they are deleted, and AR441, where *hcnC* is frameshifted.

### Exolysin

The genes for exolysin, *exlA* and its exporter *exlB* (PSPA7_4642 and PSPA7_4641, respectively), are present in all *P. paraeruginosa* as well as *P. aeruginosa* outlier group 5 (13), where their presence was reported in CF_PA39 (25) and related strains (26). Group 5, represented by the first reported complete genome, CMC-115 (27), is clearly *P. aeruginosa* but also lacks the T3SS. Exolysin production has been correlated with increased antibiotic resistance (28).

The mechanism of ExlA and ExlB has been described in detail (29). The expression of *exlBA* was found to be regulated by the Cro/CI-like repressor ErfA and the CRP-like activator Vfr (30). These two genes are ubiquitous in *P. paraeruginosa*. However, PA7 has a frameshifted *vfr* gene directly affecting *exlBA* expression (31).

### Type 2 secretion system

All *P. paraeruginosa* strains have—in addition to the well-characterized Xcp and Hxc type 2 secretion systems (T2SS) of *P. aeruginosa*—a third T2SS, called Txc (PSPA7_1407–PSPA7_1417), involved in the secretion of the chitin-binding protein CbpE (PSPA7_1419) (32). Only the Xcp system has been shown to be involved in virulence via secretion of several specific effectors (33). The Txc T2SS is encoded by genomic island RGP69 (genes PSPA7_1407–PSPA7_1420) and regulated by a two-component system (PSPA7_1420-21). Txc homologs are also found in *P. aeruginosa* outlier groups 5 and 4 (13), the latter represented by the complete genome of PA-VAP-4 (CP028368) (34). The Txc proteins of these outliers share 73.8% to 94.1% identity with their PA7 homologs (Table S1). Within *P. paraeruginosa*, the Txc systems are complete with few exceptions. In CLJ1, the *txcY* gene, encoding general secretion pathway protein L, contains a frameshift, as does the *txcS* gene, encoding general secretion pathway protein F in strain ARLG-10516, which likely leads to non-functional Txc systems in these two strains. Also, the two-component sensor gene *ttsS* is frameshifted in strain AR441 (complete genome CP029093). In other draft genomes, a few contig ends occur in this region, so the list is not exhaustive. The putative incomplete Hpl T2SS, encoded by PA2669–PA2677 in PAO1, is absent in all *P. paraeruginosa* strains.

### Glycosylation of type IV pili

A set of genes encoding the biosynthesis of the α1,5 linked D-arabinofuranose glycan, which post-translationally modifies the type IVa pili, was described in strains PA7 and Pa5196 by Harvey et al. (4). PA7 genes PSPA7_6245–PSPA7_6251 of genomic island RGP80 encode the enzymes for glycan biosynthesis. These genes occur in all *P. paraeruginosa*, but not in *P. aeruginosa*. Homologous systems were identified in *P. syringae* pv. syringae B728a and pv. *phaseolicola* 1448A. The only evident inactivating mutation found is an internal stop Q43* in the *ubiA* gene (OBG92_05927) of strain Zw26. Most *P. paraeruginosa* have group IV type IVa pilin alleles (35), composed of the pilin gene *pilA* and those encoding the oligosaccharyltransferase and accessory proteins, TfpW and TfpX. Two versions were identified: one resembling the genes in Pa5196 (ODQ13_RS09480–09475–09470) and the other resembling those in PA7 (PSPA7_5161–5160–5159) (Fig. 2). A few strains have replaced this region with a *P. aeruginosa* pilin cassette containing *pilA* and the accessory protein gene *tfpO*, as seen in strain CR1 (B7D75_23305–23300) (Fig. 2), via horizontal transfer. The occurrence of these heterologous pilin cassettes does not strictly correlate with the phylogenetic tree, suggesting multiple horizontal transfer events adjacent to, but not part of, RGP60. The glycosylation of pili renders *P. paraeruginosa* resistant to certain phages (4, 5).

### Quorum sensing

*P. aeruginosa* has three quorum sensing (QS) systems, namely *las*, *rhl*, and *pqs*. The interactions among these systems have been described (36). While LasR has been considered a master regulator, a recent publication (36) calls this concept into question and indicates that *lasR* mutants are common in *P. aeruginosa*. Thirteen of the 82 strains of *P. paraeruginosa* have mutant *lasR* genes (Fig. 2), with frameshifts (pae832, 515477, MAZ105, and ESP077), internal stops (PSA01132, EML528, WH-SGI-V-07287, and LMG5031), deletions (WH-SGI-V-07064, AZPAE14941, and PSA00066), or IS insertions (PSA00730 and PSA00554). The deletion in WH-SGI-V-07064 also inactivates the *lasI* gene. All other strains are wild type for this gene. An additional six strains (Fig. 2) have an E196D mutation presumed to be responsible for a functional defect in LasR (37). The *lasB* gene encoding elastase is intact in all strains. The *mvfR* gene, which encodes a regulator of quorum sensing (38), is frameshifted in PA7 (1), the four Argentinian strains, three closely related strains, and the more distant MIN-137 (Fig. 2). Most *P. paraeruginosa* strains lack the five amino acids DEELK at positions 150–154 in PAO1. The effect of this deletion on phenotype is unknown. Several PA7-like strains have been reported to have defective Pqs systems (39)

The *vqsM* gene was also considered a “master regulator” of quorum sensing (40, 41). This gene is present in 59 of the 82 strains (Fig. 2), and thus not ubiquitous in *P. paraeruginosa*. It is located in a region of genomic plasticity, RGP65, one of the novel RGPs in PA7 (1). This gene has an anomalous codon usage compared to the core genome, suggesting its acquisition by a recent horizontal gene transfer event.

### Serotypes

Most *P. paraeruginosa* strains are serotype O12. A strain similar to PA7 is believed to be the origin of serotype O12 in epidemic strains of *P. aeruginosa,* such as ST111, ST244, and five other sequence types. Serotype O12 *P. aeruginosa* strains typically exhibit high levels of antibiotic resistance (42). These strains contain varying lengths of DNA of *P. paraeruginosa* origin adjacent to the serotype locus RGP31 (42). Fifty-five of the 82 strains are serotype O12, including PA7 and the four related Argentinian strains. Thirteen strains contain a mixture of O11 and O12 genes. These include Zw26 and Pa5196, which are phenotypically O11. Four strains are O11. Six are O1-like (87% identity and 97% coverage), two are O4-like (82% identity and 69% coverage), and two are O8-like (98% identity and 82% coverage) (Table S1). Our BLAST results show that strains with each of these three novel serotypes are common in *P. aeruginosa*.

### TIR effector

A molecular chaperone, Tir, which mediates immune evasion (43), is encoded by a prophage gene PSPA7_2375 in genomic island RGP56. Its distribution in *P. paraeruginosa* is very limited. In addition to PA7, it is only found in three of the four closely related Argentinian strains (pae802, pae815, and pae832), as well as in strain WH-SGI-V-07370.

### Carbapenem resistance

**Fig 3:**
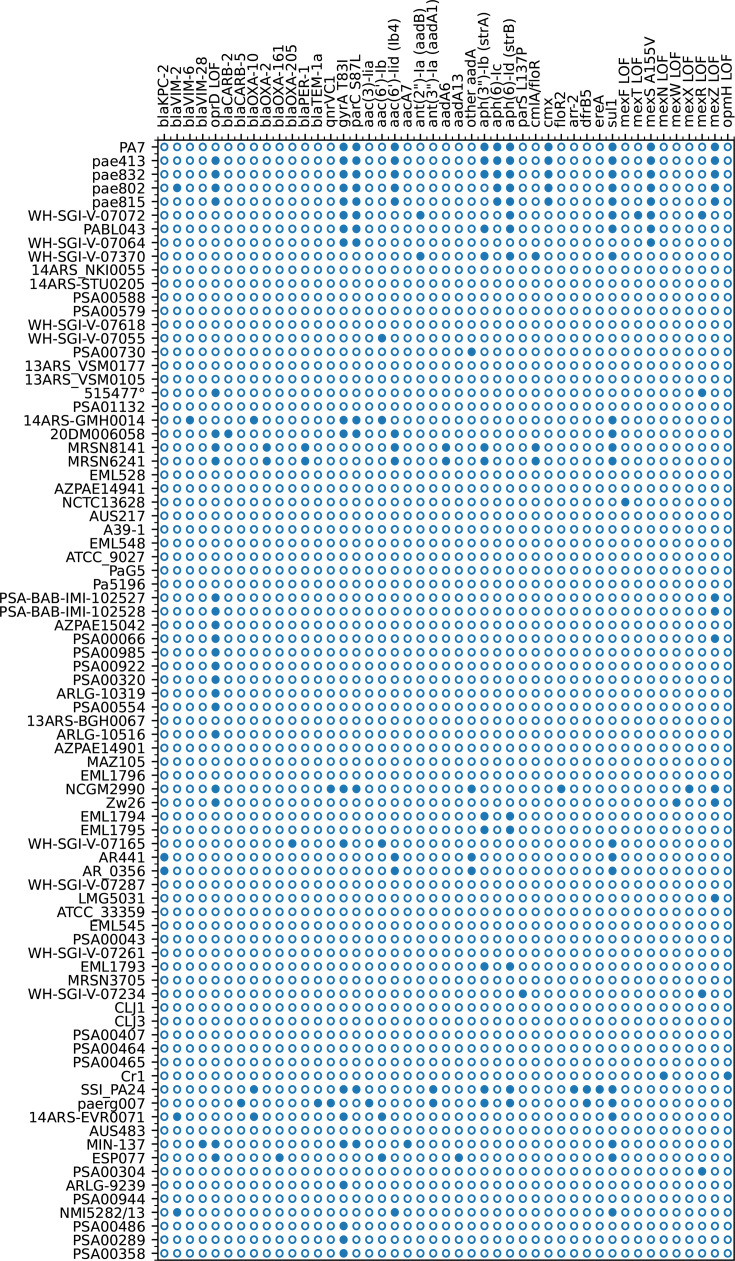
Summary of antibiotic resistance. Some of the more common point mutants associated with resistance (20, 47) are included. The filled circles indicate strains with the characteristics shown above. LOF=loss of function.

Carbapenem resistance in *P. aeruginosa* is most often due to mutations in the *oprD* gene that lead to decreased drug uptake (44). While PA7 has a wild-type *oprD* gene and is carbapenem-sensitive, the *oprD* of our four Argentinian draft strains contains frameshifts, making them phenotypically carbapenem-resistant. Minimal inhibitory concentrations (MICs) of these strains are shown in Table 3. The *oprD* gene in Zw26 is inactivated by an insertion of IS*Pa16*, while that of Pa5196 is intact. Several other strains have frameshifts or internal stops (Fig. 3). A few have a small deletion, not known to be correlated with resistance, near the carboxyl end.

**TABLE 3:** Minimal inhibitory concentrations (MICs) for the four Argentinian strains whose draft genomes are reported in this work

(µg/mL)	pae413	pae802	pae815	pae832
Piperacillin	256	256	256	>512
Ceftazidime	128	64	128	128
Cefoperazone	16	16	>32	>32
Cefotaxime	>32	>32	>32	>32
Cephalothin	>16	>16	>16	>16
Aztreonam	64	64	64	64
Imipenem	32	16	16	64
Meropenem	8	8	4	16
Ciprofloxacin	128	32	64	>128
Levofloxacin	16	8	16	>32
Amikacin	16	16	16	>32
Chloramphenicol	256	256	256	256

Carbapenemases are present in some *P. paraeruginosa* strains. Strain pae802 has plasmid pDCPR1, which was also independently isolated from *Serratia marcescens* 68313 (45), encoding *bla*_VIM-2_. Strains 14ARS-EVR0071 and NMI5282_13 also have *bla*_VIM-2_. Strain 14ARS-GMH0014 has *bla*_VIM-6_, while strain MIN-137 has *bla*_VIM-28_. The plasmids of strains AR_0356 and AR441 encode *bla*_KPC-2_. Other carbapenemases (IMP, NDM, GIM, SIM, SPM, and OXA-48) were not found (Fig. 3).

### Other resistance mechanisms

Antimicrobial resistance genes in *P. paraeruginosa* are summarized in Fig. 3. The chromosomal *bla*_OXA-50_ and *ampC* genes are ubiquitous. The *ampR* gene is frameshifted in Zw26. No *P. paraeruginosa* strains have point mutations (D135N or G154R) phenotypically identified as contributing to enhanced resistance (46). Broad-spectrum *bla*_PER-1_ is found in MRSN-6241 and MRSN-8141 (20). Narrow-spectrum *bla*_TEM-1a_ is found in one strain, *bla*_OXA-10_ in three strains, and *bla*_CARB-2_ in two strains, while *bla*_OXA-2_, *bla*_OXA-205_, and *bla*_CARB-5_ are found in one strain each (Fig. 3). The mutations *pbpC* A104P and *pbpG* V305M, correlated with beta-lactam resistance (20), are present in all strains.

The common QRDR fluoroquinolone resistance mutations *gyrA* T83I and *parC* S87L are found in PA7, the four Argentinian draft genomes, and three closely related strains as well as a few others (Fig. 3). Other point mutations in *gyrA*, *nalC*, *parC*, and *parE* are absent. The *qnrVC1* gene is found in two strains, while the *qnrA1* gene is absent in all.

The chromosomal *aph*(3′)-IIb gene (PSPA7_0973) is ubiquitous, as is *arr* (PSPA7_2339). Other aminoglycoside resistance genes are summarized in Fig. 3. None of the point mutations in *armZ*, *fusA1*, and *parR* identified as statistically significant for aminoglycoside resistance (47) are present. A single strain has the *parS* L137P mutation linked to aminoglycoside resistance.

The *catA* chloramphenicol resistance gene (PSPA7_4187) is ubiquitous in *P. paraeruginosa*. The *catB7* gene (PSPA7_4802) is frameshifted in PA7 and all other *P. paraeruginosa* strains. A CmlA/FloR family chloramphenicol efflux MFS transporter is encoded by MRSN6241 and MRSN8141. A distinct MFS transporter, *cmx* (PSPA7_6428), is found only in PA7 and the four Argentinian draft genomes. A third MFS transporter, *floR2*, is found only in strain NCGM2990.

The trimethoprim resistance gene *dfrB5* is present in two strains, while *dfrA1* and the fosfomycin resistance gene *fosA* are absent in all strains. The rifampicin resistance gene *arr-2* and the erythromycin esterase gene *ereA* are found in a single strain, SSI_PA24. The sulfonamide resistance gene *sul1* is found in several strains containing integrons (Fig. 3).

### Efflux systems

The efflux genes *mexAB-oprM*, *mexEF-oprN* and its regulator *mexT*, and *mexHI-opmD* are intact in all strains, except NCTC13628, where *mexF* is frameshifted, and WH-SGI-V-07072, where *mexT* has an internal deletion. The *mexEF-oprN* operon is up-regulated in PA7 due to an A155V mutation in *mexS,* resulting in increased fluoroquinolone resistance (48). The same mutation is found in seven strains closely related to PA7: pae413, pae832, pae802, pae815, WH-SGI-V-07072, PABL043, and WH-SGI-V-07064 (columns F–L in Table S1). These strains are human isolates and are grouped in a distinct branch of the phylogenetic tree (Fig. 1). The *mexCD-oprJ* system is deleted in one strain, Pa5196. The *mexMN* and *mexVW* systems are intact in all strains, except for frameshifts in *mexN* of CR1 and *mexW* of Zw26. The *mexXY-oprA* system described in PA7 (1, 49) is similar in all strains, except NCGM2990, where *mexX* contains an insertion containing a stop codon. Some strains have loss-of-function mutations in *mexR* and *mexZ* (including PA7 and Zw26 for the latter). These differences are summarized in Fig. 3. None of the efflux system point mutations linked to aminoglycoside resistance (47) are present in *P. paraeruginosa*. Finally, *opmH* is frameshifted in CR1. The multidrug efflux SMR transporter (PSPA7_5725) is present in all strains.

### Integrons

Class 1 integrons are found in 24 strains, and their contents are summarized in Table 4. Notably, five strains have gene cassettes encoding VIM beta-lactamases. Among them, pae802 contains a plasmid identical to that previously reported in *Serratia marcescens*, pDCPR1 (45). Some integrons, including that of PA7, have a deletion of *attI* and *qacE* and thus have no gene cassettes. These probably originate from the *aadB-*containing integron found in the closely related strain WH-SGI-V-07370. Most of the integrons have a 3′-conserved sequence containing *sul1*, but others have the alternate 3′-CS *tniRQBA* first found in Tn*402* and more recently in several carbapenemase-encoding integrons (50).

**TABLE 4:** Integron contents of *P. paraeruginosa* strains^*a*^^,^^*b*^

Strain	Accession number	5′-CS	Cassette array	3′-CS	Comments
PA7	CP000744	*intI1 attIΔ*	No cassettes	*sul1* orf5Δ	Possibly a deletion of WH-SGI-V-07370 integron
pae413	JAOYTE010000034	*intI1 attIΔ*	No cassettes	*sul1* orf5Δ	
pae832	JAPHNP010000117	i*ntI1 attIΔ*	No cassettes	*sul1* orf5Δ	
pae802 plasmid	NZ_JAPHNN010000169	*intI1*	*bla*VIM-2 a*ac(6′)-IId*	*tniRQBA*	Tn*402*-type 3′-CS
pae802	JAPHNN010000060	*intI1 attIΔ*	No cassettes	*sul1* orf5Δ	
pae815	JAPHNO010000064	*intI1 attIΔ*	No cassettes	*sul1* orf5Δ	
WH-SGI-V-07272	LLLP01000037	*intI1Δ*	No cassettes	*sul1* orf5Δ	
WH-SGI-V-07272	LLLP01000044	*intI1*	*aadB*	*traM*	Neither *sul1*-type nor Tn*402*-type 3′-CS
PABL043	QVCZ02000118	*intI1 attIΔ*	No cassettes	*sul1* orf5Δ	
WH-SGI-V-07370	LLNU01000093	*intI1*	*aadB*	*qacEΔ1 sul1* orf5Δ	Possible ancestor of PA7 integron
MRSN8141	RXTD01000086	*intI1*	unknown	unknown	*intI1* on short contig; possibly >1 integron in strain
MRSN6241	RXTL01000080	*intI1*	unknown	unknown	*intI1* on short contig; possibly >1 integron in strain
20DM006058	DAFOAA010000053	*intI1Δ*	*bla*CARB-2 *aac(6')-IId qacF*	*qacEΔ1 sul1* orf5	
14ARS-GMH0014	DAFSFU010000036	*intI1Δ*	*catB3 bla*VIM-6 *aac(6')-Ib3 bla*OXA-10	IS*110* family *tnp qacEΔ1 sul1* orf5	
WH-SGI-V-07055	LLKZ01000041	*intI1*	HP *aac(6')-IId*	*tniRQBA*	Tn*6604*/Tn*402* hybrid 3′-CS
NMI5282/13	JAFFTP010000024	*intI1Δ*	*qacL*	none	Adjacent to following integron
NMI5282/13	JAFFTP010000024	*intI1Δ*	*aac(6′)-IId bla*VIM-2	*qacEΔ1 sul1* orf5	
Paerg007	UWXD01000002	*intI1*	*qnrVC1* HP *dfrB5 aadA1*	*qacEΔ1 sul1* orf5	Two identical integrons 78 kb apart; HP may be VCR cassette
14ARS-EVR0071	DAFSFW010000021	*intI1*	catB3 blaVIM2 *aac(6')-Ib3 bla*OXA-10	IS *tnp qacEΔ1 sul1* orf5	
MIN-137	JAJBHR010000002	*intI1*	*bla*VIM-28 *aac(6′)-Il smr*	IS *tnp qacEΔ1 sul1* orf5 t*niBΔ tniA*	
ESP077	DAHOAI010000026	*intI1*	*bla*OXA-161 *aac(6′)-Ib3 aadA13*	*qacEΔ* sul1 *tniBΔ tniA*	
AR_0356 plasmid	NZ_CP027170	*intI1*	*aac(6′)-IId aadA*	*qacEΔ1 sul1* orf5	
AR441 plasmid	NZ_CP029094	*intI1*	*aac(6′)-IId aadA*	*qacEΔ1 sul1* orf5	
WH-SGI-07165	LLLU01000049	*intI1*	*aac(6′)-Ib bla*OXA-205	*qacEΔ1 sul1* orf5	
NCGM2990	DAFQBL010000010	*intI1*	*qnrVC1* HP *aadA*	*qacEΔ1 sul1 floR2*	
SSI-PA24	JAWLON010000026	*intI1*	*dfrB5 arr-2 ereA bla*OXA-10 *aadA1*	*qacEΔ1*	

^*a*^ The vestigial integrons of PA7 and five other strains, which have a deletion of *attI* and *qacE* and thus no gene cassettes, probably originate from the *aadB-*containing integron found in the closely related strain WH-SGI-V-07370.

^*b*^ Empty cells indicate no comments.

## DISCUSSION

The PA7 clade of *P. aeruginosa*, also known as group 3 (13), is now considered a distinct species, *P. paraeruginosa*, based upon diagnostic criteria, such as average nucleotide identity (ANI) and average protein identity (API) (12). We used a 200 nt sequence from the *dnaA* gene as a discriminator to identify members of this species. The genes for the arabinosylation of pili, first found in Pa5196 (2, 3), are also a discriminator, being present in *P. paraeruginosa* but absent in *P. aeruginosa*. Rudha et al. (12) used a conserved insertion into the *dgcB* gene as a marker, which is present in all *P. paraeruginosa*, but since this insertion is also found in *P. aeruginosa* group 4, it is not discriminatory. The genes for exolysin and for the Txc T2SS, while ubiquitous in *P. paraeruginosa* strains, are also found in *P. aeruginosa* taxonomic outliers, the former in group 5 and the latter in groups 5 and 4. Many *P. paraeruginosa* isolates are still annotated as *P. aeruginosa* in GenBank and RefSeq. We provide a database to permit rapid searches of all strains and contigs of *P. paraeruginosa* (see “ADDITIONAL FILE” below and the legend of Table S1).

The phylogenetic tree of *P. paraeruginosa* strains (Fig. 1) confirms that there are two sub-clades (11): one containing PA7 and the four strains closely related to PA7 whose draft genomes we report here, and the other containing Zw26, the first complete genome of a cystic fibrosis *P. paraeruginosa* isolate. This sub-clade also contains our draft genome of Pa5196, the draft genome of the *P. paraeruginosa* type strain DSM1128 (ATCC9027), and the complete genome of CR1 (11). Several genome deletions are characteristic of this sub-clade. However, the CF strain Zw26, unlike several *P. aeruginosa* CF strains, does not contain any additional genome deletions. An examination of the GenBank files of all *P. paraeruginosa* reveals a spectrum of human and environmental origins, similar to that of *P. aeruginosa* (Table S1).

PA7 was noted for its antibiotic resistance (1). Many *P. paraeruginosa* strains are resistant to carbapenems, mostly due to loss-of-function mutations in *oprD*. Two of the three *P. paraeruginosa* in the 100-strain panel of Lebreton *et al*. (20), MRSN6241 and MRSN8141, are characterized as extremely drug-resistant (XDR) (20). Many *P. aeruginosa* strains, e.g., ST235, are also multiresistant. The spectrum of resistance is similar in the two species. Interestingly, several strains of *P. paraeruginosa* encode VIM or KPC carbapenemases. The *bla*_VIM_ genes are in integrons that can be either chromosomal or, as in pae802, on a broad-spectrum plasmid also found in *Serratia* (45). The *bla*_KPC-2_ are located on plasmids unnamed 3 from AR441 and unnamed 2 from AR_0356. These are IncP-2 megaplasmids similar to the *bla*_IMP-9_ plasmid pOZ176 of *P. aeruginosa* 96 (51) and the pBT2436 family (52). The overall level of similarity, the environmental/range overlap, and the evidence of possible HGT in both *P. aeruginosa* and *P. paraeruginosa* suggest that these species can exchange carbapenemase plasmids, other plasmids, and mobile genetic islands. It is noteworthy that two strains (pae802 and MIN-137) have both a carbapenemase and an *oprD* mutation. Their effects may be additive. The three strains closest to pae802 lack the plasmid and carbapenemase. It is likely that the *oprD* mutation occurred before acquisition of the carbapenemase plasmid in pae802. The MIN-137 carbapenemase is also plasmid-mediated.

Most RGPs occur in both *P. paraeruginosa* and *P. aeruginosa*, and their contents may be shared or occur in different RGPs. Some RGPs and smaller inserts (1–3 genes) are characteristic of *P. paraeruginosa*. Among the former are the genes for arabinosylation of type IV pili in RGP80 and for ectoine transport and utilization in RGP8. Homologs of the latter are found in *P. citronellosis*, but not in *P. aeruginosa*. Genome insertions such as ICEs, Dit islands, and CRISPR-CAS systems occur in both species. In *P. paraeruginosa*, they occur in multiple clonal groups of related strains spread across the phylogenetic tree (Table S2, Fig. 1). The same is true of the type IV pilin alleles (Fig. 2). The occurrence of these multiple groups indicates a significant role for horizontal gene transfer among strains.

The genes for exolysin and its regulator (PSPA7_4641–4642), as well as for *oprA* (PSPA7_3271), are shared with group 5 outliers of *P. aeruginosa*. The percentage of similarity between these genes of *P. paraeruginosa* and their *P. aeruginosa* group 5 homologs is comparable to that of the core genomes, indicating an early acquisition and a long period of evolution. The genes for the additional Txc T2SS in RGP69 are found in *P. paraeruginosa* and in both group 5 and group 4 outliers of *P. aeruginosa*. Surprisingly, there is only about 85% similarity between the group 5 and group 4 versions. This would argue in favor of separate acquisition rather than a single acquisition followed by a loss in the common ancestor of groups 1 and 2 (see the evolutionary schema in Fig. 3C and D of Freschi et al. [13]).

Most *P. paraeruginosa* have arabinosylated pilins, but some have a *P. aeruginosa-*type pilin acquired by HGT. These HGT events emphasize that a combination of traits should be considered for definitive identification of *P. paraeruginosa* vs. *P. aeruginosa*. The identification of such strains also emphasizes the shared niches and genetic similarity of the two species that enable such exchanges to take place. Despite the switch in pilin genes in these chimeric strains, they retain the D-Ara*f* biosynthetic gene cluster, confirming that they are *P. paraeruginosa* strains that have acquired *P. aeruginosa* pilin genes. The glycosylation of pilins renders *P. paraeruginosa* resistant to certain phages (4, 5), an important consideration in designing antipseudomonal phage therapy when given the limited antibiotic options available for MDR/XDR strains.

*P. paraeruginosa* and the group 5 outliers of *P. aeruginosa* use an alternative virulence strategy. They lack the T3SS gene cluster and its effectors *exoS*, *exoT*, *exoU*, and *exoY*, whose genes are not clustered and whose loss represents independent events. These strains have an exolysin instead of a T3SS (8, 26). Genes involved in the control of exolysin have been identified (30), but the exact nature of the control of exolysin levels remains to be investigated. It is probable that a common ancestor of *P. aeruginosa* and *P. paraeruginosa* carried exolysin genes and that *P. paraeruginosa* and group 5 *P*. *aeruginosa* diverged before the loss of exolysin and the acquisition of the T3SS by a common ancestor of groups 1, 2, and 4 *P*. *aeruginosa*. Since the components of the T3SS have been proposed as candidate antigens (53, 54), the lack of a T3SS in these strains has important implications for vaccine development. While a T3SS-directed vaccine would be useful, it could also result in an increased frequency of infections with *P. paraeruginosa* and group 5 *P*. *aeruginosa* relative to T3SS-producing *P. aeruginosa*.

## MATERIALS AND METHODS

Sequencing of Zw26 was done using Illumina MiSeq and PacBio reads and assembled with Spades. Sequencing of strains pae413, pae802, pae815, pae832, and Pa5196 was done using Illumina MiSeq and assembled with Ray (pae413) or Spades (the other four strains). In this study, we employed the bacterial-annotator software (https://github.com/zorino/bacterial-annotator) with the DIAMOND aligner to comprehensively assess the *Pseudomonas paraeruginosa* strains. The strains were compared based on their coding sequence homology using a genome comparison matrix. This matrix captured the degree of similarity between all genomes and the reference PA7 strain sequences, providing insights into genetic conservation and variations among the strains. Throughout the analysis, a rigorous approach was maintained, with a threshold of 50% identity at protein level and 80% coverage over their alignments. To ensure unbiased evaluation, masking was disabled in the DIAMOND aligner, eliminating potential distortions in the genomic representation. Additionally, a phylogenetic tree was constructed with the FastTree software (v2.1.11), and 100 bootstraps using 3,177 homologous protein coding sequences conserved across all the analyzed genomes. Each of the amino acid sequences was individually aligned with MAFFT (v7.222) before being merged into a single aligned sequence. That single aligned sequence was fed into FastTree to produce the final phylogenetic tree. This statistical analysis technique enabled the assessment of the stability and reliability of the inferred relationships, contributing to a more robust understanding of the evolutionary relationships among the strains. By integrating these methodologies, our study offers a comprehensive understanding of the genetic diversity, relationships, and functional conservation within the *Pseudomonas paraeruginosa* strains. Antibiotic resistance and virulence factor genes identified in PA7 (1), along with loss-of-function and known point mutations (20), were analyzed for all strains using a Unix version of Tblastx (55) and a local database of all contigs of all *P. paraeruginosa* strains as target. The local database is being made available on Figshare https://figshare.com/articles/dataset/p_paraeruginosa_allstrains_allcontigs_fasta/26882425?file=48904948 to permit rapid searches. Integrative conjugative elements were found in complete genomes using ICEfinder (56) and in draft genomes by homology to those in complete genomes.

The six genomes reported here are available through NCBI under BioProject accession number PRJNA884324. The complete genome of *P. paraeruginosa* Zw26 has been deposited in GenBank/ENA/DDBJ under accession number CP109657. Its Sequence Read Archive accession number is SRX17828513. Draft Whole Genome Sequence accession numbers are as follows: pae413, JAOYTE00000000; pae802, JAPHNN00000000; pae815, JAPHNO00000000; pae832, JAPHNP00000000; Pa5196, JAOWAL00000000.

## Data Availability

The database file containing the sequences of all 7 complete genomes and all contigs of 75 draft genomes of the novel species *Pseudomonas paraeruginosa* can be found here: https://figshare.com/articles/dataset/p_paraeruginosa_allstrains_allcontigs_fasta/26882425?file=48904948

## SUPPLEMENTAL MATERIAL

Table S1 Legend jb.00149-25-s0001.docx Legend for Table S1.

Table S2 legend jb.00149-25-s0002.docx Legend for Table S2.

Table S1 jb.00149-25-s0003.xlsx Genomes of *P. paraeruginosa* compared to PA7

Table S2 jb.00149-25-s0004.xlsx Large genome insertions in *P. paraeruginosa* strains

ASM does not own the copyrights to Supplemental Material that may be linked to, or accessed through, an article. The authors have granted ASM a non-exclusive, world-wide license to publish the Supplemental Material files. Please contact the corresponding author directly for reuse.
