# Supplementary material for: Comparative genomics of Pseudomonas paraeruginosa
Source: J Bacteriol. 2025 Jul 25;207(8):e00149-25. doi: 10.1128/jb.00149-25 (PMC12369383; doi:10.1128/jb.00149-25)
Supplement: Table S1 Legend — Legend for Table S1. [file jb.00149-25-s0001.docx]

**Table S1** Contents of *P. paraeruginosa* strains compared to PA7. GenBank or RefSeq locus tags of homologous genes are shown. Comparisons are shown as % similarity | fraction_query_coverage | fraction_subject (PA7) coverage. Strains submitted to GenBank before Nov. 15, 2023, are included. The order of columns for the *P. paraeruginosa* strains is according to the phylogenetic tree (Figure 1) with the PA7 clade (yellow) bottom to top followed by the CR1 clade (blue) top to bottom. RGPs, AMR genes, and virulence genes are thus grouped in related strains. Representative strains of *P. aeruginosa* groups 1 (PAO1), 2 (PA14), 4 (PA-VAP-4) and 5 (CMC-115) are also included. Some additional *P. paraeruginosa* strains were omitted due to poor sequence quality, insufficient assembly (more than 250 contigs >500 nt), or lack of annotation. RGP's are highlighted; genes flanking RGP's are included to indicate RGP's that are empty in PA7. The absence of an entry for a given gene in a given strain does not necessarily signify absence of the gene; only that the above criteria are not met. This may be due to annotation errors by submitters or by PGAP. This file is available in Excel format to permit editing (e.g. regrouping strains) by the reader. Since many *P. paraeruginosa* strains are still called *P. aeruginosa* in GenBank, and searches of wgs using the genus *Pseudomonas* are very slow on the NCBI server, we provide on Figshare a file of sequences of all *P. paraeruginosa* strains and contigs: https://figshare.com/articles/dataset/p_paraeruginosa_allstrains_allcontigs_fasta/26882425?file=48904948

that allows rapid searches using tools such as tfastx36 [6] among others.
