# Supplementary material for: Comparative genomics of Pseudomonas paraeruginosa
Source: J Bacteriol. 2025 Jul 25;207(8):e00149-25. doi: 10.1128/jb.00149-25 (PMC12369383; doi:10.1128/jb.00149-25)
Supplement: Table S2 legend — Legend for Table S2. [file jb.00149-25-s0002.docx]

**Table S2** Large genome insertions in *P. paraeruginosa* strains. These include three types of integrative conjugative elements (ICE), CRISPR-Cas systems, and Dit islands.
